# Shiga Toxin–producing *Escherichia coli*, New Mexico, USA, 2004–2007

**DOI:** 10.3201/eid1508.08151515

**Published:** 2009-08

**Authors:** Sarah Lathrop, Karen Edge, Joseph Bareta

**Affiliations:** University of New Mexico, Albuquerque, New Mexico, USA (S. Lathrop, K. Edge); New Mexico Department of Health, Santa Fe, New Mexico, USA (J. Bareta)

**Keywords:** Escherichia coli, Shiga toxin, foodborne diseases, enteric diseases, bacteria, New Mexico, USA, dispatch

## Abstract

Sporadic infection with Shiga toxin–producing *Escherichia coli* (STEC) in New Mexico increased from 0.9 cases per 100,000 population (95% confidence interval [CI] 0.5–1.36) in 2004 to 1.7 (95% CI 1.14–2.26) in 2007. Non-O157 STEC was more common in nonwhite residents, children <5 years of age, and urban residents.

The epidemiology of infections and hemolytic uremic syndrome (HUS) caused by Shiga toxin–producing *Escherichia coli* (STEC) O157:H7 are well described (*1*–*4*). Non-O157 STEC infection also is associated with severe illness and HUS but often is underdiagnosed and less well understood (*3*–*7*). Studies in Europe indicate that non-O157 STEC infections occur more frequently than do STEC O157 infections (*3*). STEC O157 infection has been a notifiable disease in the United States since 1994, but non-O157 STEC infection became reportable only in 2000 (*8*). To understand trends in STEC O157 and non-O157 infections and their epidemiology in New Mexico, we analyzed population-based data from the active surveillance of clinical laboratories. This surveillance was performed as part of the Centers for Disease Control and Prevention’s (CDC’s) Foodborne Diseases Active Surveillance Network (FoodNet), to which New Mexico began contributing data in 2004.

## The Study

All STEC isolates or broths were sent for confirmation to the Scientific Laboratory Division of the New Mexico Department of Health, where Shiga toxin expression was confirmed by enzyme immunoassay (EIA); the broth was then cultured and preliminarily serotyped. Pulsed-field gel electrophoresis was performed on all isolates. The laboratory submitted non-O157 STEC isolates to CDC for additional serotyping and PCR testing for toxin genes.

This analysis comprised only sporadic cases of STEC. Negative binomial regression models were used to calculate incidence rates and assess differences in risks for STEC O157 and non-O157 infections. Variables were reporting year, patient age, race/ethnicity, sex, and rural versus urban residence (*8*); p<0.05 was considered statistically significant.

During 2004–2007, New Mexico FoodNet identified 111 cases of laboratory-confirmed sporadic STEC infection; 40 (36%) were STEC O157, and 71 (64%) were non-O157 STEC (Table 1). Six additional cases were outbreak associated. Incidence increased from 0.93 cases per 100,000 population (95% confidence interval [CI] 0.5–1.36) in 2004 to 1.07 per 100,000 (95% CI 0.61–1.52) in 2005 and 1.84 per 100,000 (95% CI 1.25–2.44) in 2006. The rate fell slightly in 2007, to 1.70 per 100,000 (95% CI 1.14–2.26) population, resulting in a test of trend that approached statistical significance (p = 0.09). From 2004 through 2007, sporadic STEC infections increased 94%. A total of 18 STEC serotypes were identified during this time. The primary serotypes responsible for the increase were STEC O157, O26, O111, and O103, constituting 75% of all STEC cases reported. Incidences of STEC serotypes O26, O111, and O103 combined increased 300% from 2004 through 2007. The proportion of non-O157 STEC ranged from 52% in 2005 to 74% in 2007.

**Table 1:** Demographic characteristics of case-patients who had laboratory-confirmed STEC infections, New Mexico, USA, 2004–2007*

Characteristic	No. case-patients		
	O157 STEC	Non-O157 STEC	Total
Age group, y†			
<1	3	3	6
1–4	3	26	29
5–10	10	7	17
11–18	4	12	16
19–29	5	7	12
30–39	4	2	6
40 –49	1	2	3
50–59	1	5	6
>60	9	7	16
Sex			
Male	18	34	52
Female	22	37	59
Race/ethnicity			
White non-Hispanic	17	17	34
White Hispanic	11	12	23
White, unknown ethnicity	5	4	9
Native American	3	10	13
African American	1	0	1
Other	0	5	5
Unknown	3	23	26
Type of county			
Urban	29	64	93
Rural	11	7	18
Year of diagnosis			
2004	7	11	18
2005	10	11	21
2006	14	23	37
2007	9	26	35
Total no. case-patients	40	71	111
*STEC, Shiga toxin–producing *Escherichia coli*; O157, serotype O157:H7. †Median age (range) of patients: STEC O157–infected, 18 y (4 mo–78 y); non-O157 STEC–infected, 10 y (5 mo–70 y); total, 13.3 y (4 mo–78 y).			

Although STEC O157 was the 1 serotype most often identified, infections caused by non-O157 STEC (all serotypes) were diagnosed more frequently. STEC O26 (18%) and O111 (13%) were the most commonly identified non-O157 STEC serotypes. Other STEC O serotypes (O103, O121, O46, O177, and O91) were responsible for 33% of all STEC infections. Most isolates were positive for toxin gene *stx_1_* (86%), intimin (66%), and enterohemolysin A (81%).

STEC O157 infection was significantly more likely to be diagnosed in adults (half of all cases, one third of non-O157 STEC cases) than in children (<18 years of age) (p = 0.01). Non-O157 STEC serotypes were most commonly identified in children 1–4 years of age. STEC O157 infections occurred most commonly in children 5–10 years of age, followed by adults >60 years. Sex distributions were similar for patients with STEC O157 and non-O157 infections (55% female vs. 45% male and 52% vs. 48%, respectively). White non-Hispanics, which constitute 43% of New Mexico’s population, made up 31% of all confirmed STEC infections in New Mexico; white Hispanics (42%) made up 21% of confirmed STEC cases; and Native Americans (10%) made up 12% of cases (Table 1).

More laboratory-confirmed STEC infections were diagnosed during the summer months than during the rest of the year. STEC O157 cases were diagnosed more frequently in September (10 cases); non-O157 STEC cases were most frequently diagnosed in June and July (9 cases each).

Although STEC O157 infections was diagnosed in more persons than were non-O157 STEC infections (28% vs. 16%), the difference was not significant. Patients with STEC O157 infections stayed in the hospital a mean of 6 days (median 4), compared with a mean of 4.5 days (median 3) for patients with non-O157 STEC infections, also not significant. All 5 reported cases of HUS were caused by STEC O157.

International travel was related to STEC infection for 12 (11%) patients. Two (5%) STEC O157 cases were travel related, as were 4 (10%) cases each of STEC O111 and O26; and 1 (1%) case each of STEC O128 and O103. Travel to Mexico was documented for 10 of the 12 travel-associated cases.

Most (99 [89%]) persons with STEC were from New Mexico’s urban counties. We calculated incidence rate ratios between STEC O157 and non-O157 infections during 2004–2007 (Table 2), while adjusting for variables that were significant in negative binomial models, including race (white non-Hispanic vs. nonwhite), age (<5 years vs. >5 years), and county (urban vs. rural). More patients with non-O157 STEC infection during this time were nonwhite, <5 years of age, and residents of urban counties. Patients infected with STEC O157 were more likely to be white non-Hispanic, >5 years of age, and residents of rural counties.

**Table 2:** Comparisons of risk for infection with STEC O157 versus non-O157 STEC types, by demographic characteristic, averaged population, New Mexico, USA, 2004–2007*

Characteristic	Unadjusted incidence rate ratio† (95% CI)	p value	Adjusted incidence rate ratio† (95% CI)	p value
Race				
Other	1		1	
White	3.21 (1.42–7.27)	0.01	3.03 (1.34–6.90)	0.008
Age, y				
<5	1		1	
>5	2.61 (1.09–6.22)	0.03	2.74 (1.15–6.54)	0.031
County				
Urban	1		1	
Rural	1.96 (0.98–3.92)	0.05	1.84 (0.91–3.69)	0.089

*STEC, Shiga toxin–producing *Escherichia coli*; O157, serotype O157:H7; CI, confidence interval. †Corrected for overdispersion by using negative binomial distribution.

## Conclusions

The data collected by New Mexico’s FoodNet surveillance network indicate that sporadic STEC cases increased substantially from 2004 through 2007. Reports of STEC O157 infection doubled from 7 to 14 from 2004 to 2006 but dropped to 9 in 2007. However, the number of non-O157 STEC cases continued to climb and accounted for most of the increase in overall STEC rates in New Mexico during this time, similar to rates in Connecticut and other FoodNet sites (*4*,*9*).

Non-O157 STEC infections ranged from 52% to 74% of all Shiga toxin–positive cases diagnosed each year and 64% of all identified STEC cases. New Mexico was second only to Colorado (2.12 cases per 100,000 population) among FoodNet sites for non-O157 STEC incidence in 2007 (*10*). Similar to serotypes reported from other locations, STEC serotypes O26, O111, and O103 made up most non-O157 infections in New Mexico (*1*), especially O26 and O111 (18% and 13% of all cases, respectively).

As previously reported (*1*), non-O157 STEC infections occurred commonly in young children in this study. They also occurred at a higher rate for non-white New Mexico residents. Another recent study similarly found higher shigellosis rates in counties with higher proportions of Hispanics (*11*). This study concurs with others that have reported finding the highest rates of STEC O157 rates in rural communities (with increased opportunities for animal contact) and in the West (*12*,*13*).

Year-to-year increases in numbers of non-O157 STEC infections, both nationally and in New Mexico, must be interpreted with caution because of changes in laboratory testing practices. Although the state’s largest clinical laboratory performed EIAs before active surveillance was implemented in 2004, other laboratories throughout the state might have changed their testing practices.

Risk factors for non-O157 STEC infections may be similar to those for STEC O157 infections (*9*), but additional studies are needed to elucidate differences and similarities between them, as well as among non-O157 STEC serotypes (*14*). Clinical laboratories should simultaneously screen for Shiga toxin and culture all positive isolates to determine the true incidence of non-O157 STEC infections.
